# RB-GAT: A Text Classification Model Based on RoBERTa-BiGRU with Graph ATtention Network

**DOI:** 10.3390/s24113365

**Published:** 2024-05-24

**Authors:** Shaoqing Lv, Jungang Dong, Chichi Wang, Xuanhong Wang, Zhiqiang Bao

**Affiliations:** 1 School of Communication and Information Engineering, Xi’an University of Posts and Telecommunications, Xi’an 710121, China; djg03192012@163.com (J.D.); dwangcc@163.com (C.W.); wxh@xupt.edu.cn (X.W.); baozhiqiang@xupt.edu.cn (Z.B.); 2 Shaanxi Key Laboratory of Information Communication Network and Security, Xi’an University of Posts and Telecommunications, Xi’an 710121, China

**Keywords:** word embedding, RoBERTa, BiGRU, text classification, multi-head GAT

## Abstract

With the development of deep learning, several graph neural network (GNN)-based approaches have been utilized for text classification. However, GNNs encounter challenges when capturing contextual text information within a document sequence. To address this, a novel text classification model, RB-GAT, is proposed by combining RoBERTa-BiGRU embedding and a multi-head Graph ATtention Network (GAT). First, the pre-trained RoBERTa model is exploited to learn word and text embeddings in different contexts. Second, the Bidirectional Gated Recurrent Unit (BiGRU) is employed to capture long-term dependencies and bidirectional sentence information from the text context. Next, the multi-head graph attention network is applied to analyze this information, which serves as a node feature for the document. Finally, the classification results are generated through a Softmax layer. Experimental results on five benchmark datasets demonstrate that our method can achieve an accuracy of 71.48%, 98.45%, 80.32%, 90.84%, and 95.67% on Ohsumed, R8, MR, 20NG and R52, respectively, which is superior to the existing nine text classification approaches.

## 1. Introduction

As a crucial task in the realm of text mining, text classification [1] seeks to organize and summarize textual data by categorizing them into predefined groups, finding applications in diverse fields such as spam detection [2], sentiment analysis [3], and news classification [4]. Traditional approaches to text classification involve the amalgamation of feature engineering with shallow classification models [5], including K-nearest neighbors [6], naive Bayes [7], and support vector machines [8]. However, these methods often require intricate feature engineering and fail to adequately account for the sequential structure of text data, thus impeding the model’s capacity to comprehend semantic relationships between words. In recent years, the advent of sequence deep learning models [9] has heralded a paradigm shift in text classification. These models eliminate the reliance on manual feature design and rule-based systems, autonomously extracting rich semantic information from vast amounts of text data. Consequently, contemporary research in text classification predominantly revolves around data-driven deep learning models, prioritizing the extraction of meaningful patterns and representations directly from the data, which has demonstrated its effectiveness in processing sequential and grid-structured text data. The most widely adopted sequence deep learning techniques include convolutional neural networks (CNNs) [10] and recurrent neural networks (RNNs) [11].

However, when faced with text graphs containing multi-level relationships among documents, paragraphs, and words that encompass diverse and rich information, sequence deep learning models may fall short and struggle to effectively handle graph-structured data [12]. On the other hand, graph neural networks (GNNs) [13] demonstrate an excellent ability to process graph-structured data and capture intricate relationships between entities, finding wide applications in recommendation systems [14], drug discovery [15], and protein design [16]. Recently, GNNs have gained attention in text classification tasks, such as sentiment analysis [17] and document classification [18], due to their significance in capturing complex relationships within unstructured text graph data. By representing text data as a graph structure, GNNs can learn the representations of nodes and edges in the text graph, thereby modeling the relationships among the text data. However, these methods also exhibit certain limitations. For instance, existing models typically use one-hot encoding to initialize node features, resulting in high-dimensional and sparse feature matrices, potentially failing to effectively express text similarity. Furthermore, current methods overlook word relationships within the context of document sequences, neglect fine-grained word interactions, and struggle to handle emotional text data. As the field advances, addressing these challenges will be crucial to further enhancing the capabilities of GNNs for text classification tasks.

Meanwhile, several pre-trained word embedding models have emerged to improve the understanding of text relationships, these include bidirectional encoder representations from transformers (BERT) [19] and robustly optimized BERT approach (RoBERTa) [20]. RoBERTa stands as an optimized iteration of the BERT language model, enhancing pre-training through the utilization of larger mini-batches and expanded datasets. Notably, RoBERTa adjusts key hyperparameters from BERT, which involves eliminating BERT’s next-sentence pre-training objective and training with significantly larger mini-batches and learning rates. Thanks to its dynamic and contextualized representations, RoBERTa offers advantages in word embedding by capturing nuanced semantic meanings that adapt to surrounding text contexts, resulting in superior performance across various natural language processing tasks. Numerous methods have been introduced to handle text data and grasp both forward and backward dependencies within sequences, such as BiLSTM and BiGRU. BiGRU, a variant of the recurrent neural network (RNN) architecture, aims to enhance the model’s ability to extract information from past and future states in a sequence. The “bidirectional” characteristic of BiGRU signifies its capability to process data in both forward and backward directions. This enables it to comprehend the context from both preceding and succeeding points, effectively addressing long-term dependencies within the sequence. Such capability proves particularly valuable in text classification tasks, where the interpretation of words often hinges on both preceding and succeeding words.

Inspired by recent advancements, and aiming to tackle the challenges in text classification tasks leveraging graph neural networks (GNNs), this paper introduces a novel model called RoBERTa-BiGRU with Graph ATtention network (RB-GAT). RB-GAT initially constructs a heterogeneous graph of text words, utilizing techniques like TFIDF. Subsequently, the RoBERTa-BiGRU architecture is employed to generate embedding representations for both texts and words, effectively preserving long-term dependencies and bidirectional information within sentences. Finally, a multi-head two-layer graph attention network (GAT) is employed to train the constructed heterogeneous graph of text words, refining the corresponding text and word representations.

RB-GAT integrates the graph attention mechanism to assess the significance of adjacent nodes in the message-passing process. This mechanism calculates weight values, allows the model to assign varying weights to different neighbor nodes, and captures intricate relationships between nodes. Moreover, the model incorporates a multi-head attention mechanism that operates based on a user-defined number of heads, which enables the model to extract text data information from diverse perspectives, enhancing the stability and robustness of the network. 

The contributions of this paper are listed as follows:

Integrating Graph Neural Networks with RoBERTa-BiGRU Architecture: The RB-GAT model leads the way in combining GNNs with the RoBERTa-BiGRU framework, leveraging the distinct strengths of each to effectively navigate the intricacies of text classification. This pioneering approach enables the model to discern and explore the intricate relationships among words and documents within text graphs, as well as the sequential dependencies inherent in sentences. As a result, it offers a comprehensive understanding of textual data.

Utilization of a Multi-Head Graph Attention Network (GAT): RB-GAT elevates text and word representation refinement beyond conventional methods by embracing a multi-head, two-layer GAT. This technique endows the model with a multi-head attention mechanism, allowing for a nuanced evaluation of the importance of neighboring nodes through variable weight assignments. This capability not only captures the subtle nuances of relationships but also significantly enhances the interpretability of the model.

Overcoming Prevalent Constraints in Text Classification Models: RB-GAT systematically tackles critical limitations that are present in existing text classification methodologies. Notably, it departs from the simplistic approach of one-hot encoding for initializing node features and rectifies the oversight of complex word interactions within document sequences. These improvements substantially enhance GNNs’ ability to analyze and interpret emotionally and contextually rich text data, representing a significant advancement in text classification research.

## 2. Related Work

### 2.1. Sequence Deep Learning-Based Text Classification Methods

Since 2010, sequence deep learning-based text classification methods have steadily supplanted traditional machine learning techniques. Numerous effective models within the realm of sequence deep learning have been proposed and widely embraced for a myriad of text classification tasks. A notable example is the recurrent neural network (RNN) [11], which treats words as temporal segments, capturing word relationships by calculating their similarities. RNNs acquire dependencies between words by memorizing preceding text and learning from subsequent text. However, RNNs encounter challenges, such as gradient explosion and vanishing gradients, attributed to long-term memory. To address these issues, the long short-term memory network (LSTM) [21] emerged as a variant of RNN, incorporating memory selection. LSTM mitigates problems like gradient explosion and vanishing gradients by utilizing forget, input, and output gates to regulate the flow of information in and out of its units. Researchers have extended LSTM for text sentiment classification. Xu et al. [22] proposed a bidirectional LSTM (BiLSTM) model, processing text sequences in both forward and backward directions using multiple LSTM networks to glean word context information. Additionally, Tai et al. [23] developed the tree-LSTM model, extending LSTM to tree-structured networks, effectively acquiring rich semantic representations, and showcasing favorable performance in sentiment classification and sentence relationship prediction tasks. Furthermore, variants of LSTM, such as the simple recurrent unit (SRU) [24], have been proposed. SRU mirrors LSTM in terms of model design but boasts faster computation speed for classification tasks, approximately 5 to 9 times swifter than LSTM, while achieving comparable or even superior classification performance. These models have sparked a revolution in text classification, enabling more precise and efficient analysis of textual data. However, these methods are limited when seeking to understand global context because they encode only a limited amount of historical information at each timestep.

Unlike recurrent neural network models, which treat text data as time series data, convolutional neural network (CNN) models take on a grid-like representation for text classification tasks [25]. While recurrent neural network (RNN) models demonstrate proficiency in understanding the semantic content within lengthy texts, CNN models specialize in identifying local features within the text, such as keywords or specific topics conveying particular emotions. Grefenstette et al. [26] introduced the dynamic convolutional neural network (DCNN) for text classification, utilizing temporal convolutions to capture variable-length phrases. Subsequently, Kim et al. [10] proposed a simpler CNN-based text classification model, employing a single convolutional layer on word vectors obtained from a neural language model. Liu et al. [27] extended the Kim CNN model architecture by introducing a hidden layer to reduce the dimensionality of the vector representation. They also incorporated the maximum pooling method to obtain a more compact document representation, thereby enhancing the model’s performance. Furthermore, Zhang et al. [28] proposed a character-level CNN model for text classification tasks that uses fixed-size characters as input and employs CNNs for feature extraction, achieving favorable results in various text classification scenarios. These CNN-based models provide alternative approaches to text classification by leveraging the grid-like structure of text data, demonstrating effectiveness in capturing local features and achieving competitive performance across various text classification tasks. However, the primary limitation of CNN-based models lies in their fixed context window size, which may hinder the capture of longer dependency patterns present in the text.

### 2.2. Graph Neural Network-Based Text Classification Methods

Graph neural networks (GNNs) are specialized neural network models crafted for processing graph-structured data, capable of representing a variety of data types such as social networks and molecular structures. These models leverage the relationships between nodes in a graph to glean more meaningful data representations. The updating of node representations within a graph is facilitated through a messaging mechanism, wherein each node aggregates information from its neighbors and adjusts its vector representation accordingly. These resulting representations find application in diverse tasks, including node classification and graph classification. In the context of text classification tasks, GNNs typically construct a text graph structure to capture intricate relationships between nodes and effectively represent the text data. For instance, Yao et al. [29] introduced TextGCN, a text graph convolutional network based on graph convolutional networks (GCN). TextGCN treats the text as a static heterogeneous graph, aggregating and updating node features using the GCN model. This approach demonstrates excellent performance on classified datasets, such as topics and news. Liu et al. [30] have presented TensorGCN, a tensor graph convolutional network tailored for text classification tasks. The model constructs three text graph structures representing semantics, syntax, and word order, respectively. GCN is harnessed in order to learn from these structures, resulting in remarkable text classification performance. Defferrard et al. [31] introduced the Graph-CNN model, a significant advancement in adapting CNNs to process graph-structured data efficiently through spectral-based convolutional operations. To address the complexity and computational demands of traditional GCN models, Wu et al. [32] proposed a simplified approach known as the simplified graph convolutional (SGC) network. This method eliminates nonlinearities between graph convolution layers and consolidates weight matrices between layers. SGC has demonstrated comparable or even superior performance on various benchmark datasets for text classification. Hu et al. [33] have proposed a heterogeneous graph attention network, named HGAT, based on the graph attention network (GAT). The HGAT model incorporates node-level and type-level two-tier attention mechanisms, proving effective for short text classification. Furthermore, Zhang et al. [34] have introduced the TextING model to address the limitations of existing GNN models in capturing relationships between contextual words in documents and completing the inductive learning of new words. In summary, GNNs offer a potent framework for processing graph-structured data and have been successfully applied to various text classification tasks, allowing for the modeling of rich relationships within textual data. However, these models primarily concentrate on extracting and utilizing the information embedded within the graph structure of the text. They often overlook the effective harnessing of a text’s semantic content, especially the profound semantic insights available from various pre-trained models.

## 3. Method

### 3.1. Model Architecture

**Figure 1:**
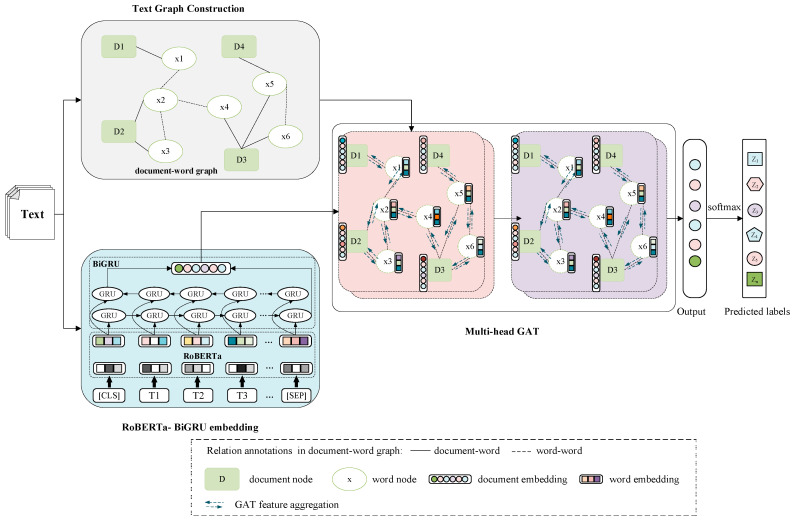
The overall framework of the RB-GAT model.

To achieve enhanced text classification performance, we propose a text classification model based on RoBERTa-BiGRU word embedding with a multi-head graph attention network (RB-GAT). As illustrated in Figure 1, the RB-GAT model comprises three main components: text graph construction, RoBERTa-BiGRU embedding, and the multi-head graph attention network. The first step involves constructing a document–word graph structure to capture contextual relationships within the text. Next, RoBERTa-BiGRU word embedding is utilized to obtain word embeddings with contextual information from the entire text. Subsequently, a multi-head GAT model is trained using the word embedding and the document–word graph. This allows for a nuanced and comprehensive analysis of the graph data, enabling the model to focus on the most relevant parts of the text for classification. Finally, the processed features are passed through a Softmax layer for text classification, enabling the model to predict the most likely category for the given text.

RoBERTa has established state-of-the-art performance in various NLP tasks due to its deep bidirectional transformer architecture, which captures rich contextual information from extensive pre-training. Its capability to comprehend nuanced language patterns and relationships within text makes it an ideal choice for generating high-quality embeddings. By leveraging RoBERTa, RB-GAT utilizes these rich embeddings, providing a robust foundation for subsequent processing. These embeddings encapsulate both semantic and syntactic nuances, essential for accurate text classification. BiGRU models are renowned for their efficiency in capturing sequential dependencies within data. Unlike unidirectional RNNs, BiGRUs process information in both forward and backward directions, thereby retaining information from both past and future contexts in a sequence. Integrating BiGRU enables RB-GAT to effectively model long-term dependencies and bidirectional contextual information within sentences, enhancing the model’s ability to understand word order and relationships, which are critical for text classification tasks. GAT introduces an attention mechanism in order to graph neural networks, allowing the model to focus on the most relevant parts of the graph. The attention mechanism assigns different weights to neighboring nodes, capturing intricate relationships and varying node importance. Employing a multi-head, two-layer GAT allows RB-GAT to refine text and word representations through nuanced evaluations of neighboring nodes. The multi-head attention mechanism provides multiple perspectives on the data, enhancing the model’s robustness and interpretability. This is particularly important for heterogeneous graphs, where the significance of connections can vary significantly. The combination of RoBERTa, BiGRU, and GAT enables RB-GAT to effectively address the complexities inherent in text classification tasks.

In the following sections, we will elaborate on each component in detail.

### 3.2. Text Graph Construction

To capture the intricate relationships and dependencies between words and documents, we constructed the text graph following a methodology akin to that employed in TextGCN [29]. We formed a heterogeneous graph G that encompasses both word and document nodes. Subsequently, edges were established between words and documents. Specifically, an edge was created if a word appeared in a document. The weight of this edge was typically determined by the term frequency–inverse document frequency (TF–IDF) of the word in that document. Additionally, edges were added between word nodes based on their co-occurrence within a specific context window within the corpus. The weight of these edges could be set as the point-wise mutual information (PMI) of the word pair, and if the PMI was less than 0, it was set to 0.

More specifically, the term frequency–inverse document frequency is calculated using the following formula:

(1) $$\mathrm{TF}-\mathrm{IDF}\left(w,\;d\right)=\mathrm{TF}\left(w,\;d\right)\times \log\left(\frac{N}{\mathrm{DF}\left(w\right)}\right)$$

where $\mathrm{TF}\left(w,\;d\right)$ is the frequency of word *w* in document *d*, *N* is the total number of documents, and DF(*w*) is the number of documents containing word *w*.

The point-wise mutual information is calculated as follows:

(2) $$\mathrm{PMI}\left(w_{i},w_{j}\right)=\log\left(\frac{p\left(w_{i},w_{j}\right)}{p\left(w_{i}\right)p\left(w_{j}\right)}\right)$$

where *p*(*w_i_*, *w_j_*) is the probability of words *w_i_* and *w_j_* co-occurring within a context window, and *p*(*w_i_*)and *p*(*w_j_*) are the probabilities of words *w_i_* and *w_j_* occurring in the corpus.

Formally, the weight of edge between node *i* and node *j* in graph *G* is defined as follows:

(3) $$\begin{array}{c} A_{ij}=\left\{\begin{array}{cc} \mathrm{PMI}\left(i,\;j\right) & \mathrm{if}\;i,j\;\mathrm{are}\;\mathrm{words},\;\mathrm{PMI}\left(i,j\right)>0\\ \mathrm{TF}-\mathrm{IDF}(i,\;j) & \mathrm{if}\;i\;\mathrm{is}\;\mathrm{word},\;j\;\mathrm{is}\;\mathrm{document}\\ 1 & \mathrm{if}\;i=j\\ 0 & \mathrm{otherwise} \end{array}\right. \end{array}$$

### 3.3. RoBERTa-BiGRU Embedding

To better capture the intricate relationships between texts, our model leverages RoBERTa and BiGRU to obtain word embedding vectors. RoBERTa, like many advanced natural language processing (NLP) models, requires tokenization as a fundamental preprocessing step for text processing. Tokenization standardizes the format of input text, ensuring consistent processing across various text data. We employ byte-pair encoding (BPE) for tokenizing the input text into tokens *T_i_*, a common practice in transformer-based models. Special tokens such as [CLS] for marking the beginning of text and [SEP] for segment separation are added to aid RoBERTa in understanding text boundaries and structure. Each token from the preprocessing step is mapped to a unique vector in a high-dimensional space. This mapping is achieved by passing the tokens through RoBERTa’s embedding layer, which retrieves the initial embedding for each token. RoBERTa employs multiple layers of transformer blocks to process these initial embeddings. Each block applies self-attention mechanisms, enabling the model to evaluate the significance of other tokens within the same text when representing a specific token. A given token’s embedding can be extracted directly from a specific layer within the model or by aggregating embeddings across multiple layers to capture a richer representation. This results in each token’s embedding encapsulating not only its own semantic information, but also contextual nuances derived from the entire text.

These word embeddings **X** = {*x*_1_, *x*_2_, …, *x_n_*} for each document are then input into the BiGRU model. The BiGRU architecture effectively captures bidirectional contextual information by leveraging the strengths of gated recurrent units (GRUs) to address issues related to long-term dependencies in sequential data. BiGRU integrates two GRU networks processing the sequence in opposite directions: one forward GRU that captures forward context (from the beginning to the end of a sequence) and one backward GRU that captures backward context (from the end to the beginning of a sequence). The final contextual embedding of the *i*-th text $\vec{h_{i}}$ is generated by concatenating its corresponding forward $h_{i}^{F}$ and backward hidden states $h_{i}^{B}$, as follows:

(4) $$\vec{h_{i}}=[h_{i}^{F}||\;h_{i}^{B}]$$

where || denotes concatenation.

The output of the BiGRU model comprises bidirectional contextual embeddings for each word in the input sequence. These embeddings are enriched with comprehensive contextual information, rendering them well-suited for downstream text classification tasks.

### 3.4. Multi-Head GAT Model

We apply GAT to process the document–word graph *G* and node attribute $h=\left\{\vec{h_{1}},\;\vec{h_{2}},\;\ldots ,\;\vec{\;h_{n}}\right\}$. Each node’s features $\vec{h_{i}}$ are linearly transformed using a shared weight matrix **W**. The attention coefficients between each pair of nodes are computed to determine how much focus should be given to a neighboring nodes’ features. The attention coefficient *e*_ij_ from node *i* to node *j* in *G* is calculated as follows:

(5) $$e_{ij}=\mathrm{LeakyReLU}\left(\vec{a}\left[W\vec{h_{i}}||\;W\vec{h_{j}}\right]\right)$$

where $\vec{a}$ is a learnable weight vector, LeakyReLU is a non-linear activation function and || denotes concatenation.

The raw attention coefficients are normalized using the Softmax function to make coefficients easily comparable across different nodes, as follows:

(6) $$\alpha_{ij}=\frac{\exp\left(e_{ij}\right)}{\sum_{k\in N_{i}}\exp\left(e_{ik}\right)}$$

where $N_{i}$ denotes the neighbors of node *i*, including *i* itself if self-loops are added.

The node features are updated by aggregating neighbors’ features weighted by the normalized attention coefficients, as follows:

(7) $$\vec{h_{i}^{\prime }}=\sigma \left(\underset{j\in N_{i}}{\sum }\alpha_{ij}W\vec{h_{j}}\right)$$

where σ denotes the ReLU non-linear activation function.

To stabilize the learning process of the model, we employ a multi-headed attention mechanism. The hidden states of the nodes are calculated using *K* independent attention mechanisms through Equation (7), and then the *K* outputs are concatenated together as the input of the next layer.

(8) $$\vec{h_{i}^{\prime }}={\left|\right|}_{k=1}^{K}\sigma \left(\underset{j\in N_{i}}{\sum }\alpha_{ij}^{k}W^{k}\vec{h_{j}}\right)$$

The process is repeated for the second layer, taking $\vec{h_{i}^{\prime }}$ (the output of the first layer) as the input. This involves applying a new weight matrix and recalculating the attention coefficients and aggregated features to produce the final output features for each node $\vec{h_{i}^{\prime \prime }}$. The embeddings of nodes (words/documents) in the second layer have the same dimensionality as the label set, and predictions are obtained by applying Softmax:

(9) $$z_{i}=\frac{e^{\vec{h_{i}^{\prime \prime }}}}{\sum_{k=1}^{C}e^{\vec{h_{k}^{\prime \prime }}}}$$

The objective of training is to minimize the cross-entropy loss between the true and predicted labels. The loss function is defined as follows:

(10) $$loss=-\overset{N}{\underset{i=1}{\sum }}y_{i}\log z_{i}$$

where *N* is the number of documents, $y_{i}$ is the actual label of the document *i*, and $z_{i}$ is the predicted label of the document.

## 4. Experiments

This section offers a detailed insight into the experimental methodology, starting with a description of the datasets employed. It then proceeds to elaborate on the experimental procedures and the evaluation criteria utilized in the study. Subsequently, a comprehensive analysis of the results obtained from the comparative assessment of various models is presented.

### 4.1. Datasets

We conducted our experiments on five widely used text classification benchmark datasets: Ohsumed, R8, MR, 20NG, and R52 [29]. A summary statistics of the benchmark datasets is presented in Table 1.

The Ohsumed dataset (http://disi.unitn.it/moschitti/corpora.htm, accessed on 12 April 2023) was sourced from the MEDLINE database, which is a significant bibliographic database of medical literature maintained by the National Library of Medicine. It consists of 7400 single-label documents, evenly distributed across 23 different disease categories, with an average document length of 135.82 words. For this study, 3357 documents were used as the training set and 4043 documents as the test set.

The R8 dataset (https://kdd.ics.uci.edu/databases/reuters21578/reuters21578.html, accessed on 22 April 2023) is a subset of the Reuters dataset. It comprises 7674 documents, equally divided into eight different categories. The dataset has an average document length of 65.72. For this paper, 5485 documents were utilized as the training set and 2189 documents as the test set.

The MR dataset (http://www.cs.cornell.edu/people/pabo/movie-review-data/, accessed on 10 April 2023) is a collection of movie reviews designed for binary emotion classification. It consists of 10,662 documents, with 5331 being positive reviews and 5331 being negative reviews. The dataset has an average document length of 20.39 words. In our experiments, 7108 documents of this dataset were employed as the training set and 3554 documents as the test set.

The 20NG dataset (http://qwone.com/~jason/20Newsgroups/, accessed on 1 May 2024) is a collection of 18,846 documents, evenly distributed across 20 different newsgroups, with an average document length of 221.26 words. Each group corresponds to a distinct topic, ranging from politics and sports to science and more. In our experiments, 11,314 documents from this dataset were used as the training set, and 7532 documents are used as the test set.

The R52 dataset (http://www.cs.cornell.edu/people/pabo/movie-review-data/, accessed on 1 May 2024) is another subset of the Reuters dataset. It comprises 9100 documents categorized into 52 classes, with an imbalanced category distribution. The average document length is 69.82 words. For our experiments, 6532 documents were employed as the training set, while 2568 documents were used for the test set.

### 4.2. Implementation Details

This study adopted Python 3.7 as the programming language and utilized Torch 1.10.2 as the deep learning framework. The GPU employed was a NVIDIA GeForce RTX 2080ti (Nvidia, Santa Clara, CA, USA). A heterogeneous graph of the original text was constructed following the approach described in TextGCN [29]. In accordance with the strategy delineated in the proposal [29], a random subset comprising 10% of the documents from the training dataset was selected to establish the validation set.

When setting the hyperparameters, we leveraged insights from prior research and experimental experience, and referred to optimization algorithms based on Bayesian methods. Additionally, we utilized the Python open-source toolkit ‘advisor’ for parameter optimization [35]. 

After experimental comparison, the model parameters were as follows: the text sequence length was set to 128, with any exceeding length truncated. For model training, a batch size of 32 was utilized, and the training was performed for 100 iterations. Early stopping was applied. The loss function employed was cross-entropy, and the optimizer used was Adam [36]. The learning rates for RoBERTa and BiGRU were set to 0.00001, the GAT module adopted a learning rate of 0.001, and a dropout rate of 0.5 was applied. To evaluate the model’s performance under various conditions, the number of attention heads was set to 2, 4, 6, 8, and 10, respectively. For the models that we compared, we maintained the parameter settings as specified in their original papers.

### 4.3. Experimental Metrics

In our framework, we employ accuracy and macro *F*1 as the primary metrics for evaluation. For each document category $y_{i}$, the methodologies for computing accuracy and *F*1 score are detailed in Equations (11)–(14).

(11) $$Accuracy=\frac{TP+TN}{TP+TN+FP+FN}$$

(12) $$Precision=\frac{TP}{TP+FP}$$

(13) $$Recall=\frac{TP}{TP+FN}$$

(14) $$Macro\;F1=\frac{2\times Precision\times Recall}{Precision+Recall}$$

where *TP* represents the count of instances within class $y_{i}$ accurately classified as $y_{i}$, whereas *FP* denotes the instances from classes distinct from $y_{i}$ yet classified as $y_{i}$. Conversely, *TN* refers to instances not belonging to $y_{i}$ and correctly classified into categories other than $y_{i}$, and *FN* embodies the instances of $y_{i}$ that have been erroneously classified into classes other than $y_{i}$.

### 4.4. Experimental Results and Analysis

#### 4.4.1. Accuracy of Different Algorithms

In the comparative experiment, three main types of models were employed: (1) word embedding-based models, such as PV-DBOW and FastText; (2) sequence deep learning-based models utilizing CNN and BiLSTM; (3) graph neural network-based models, primarily including TextGCN, TensorGCN, Graph-CNN, SGC, TextING, TextSSL [37] and MHGAT [38]. An extensive experimental evaluation was conducted using benchmark datasets.

The results outlined in Table 2 illustrate that our proposed RB-GAT model outperforms all baseline methods, including word embedding-based models, sequence deep learning-based models, and graph-based approaches. Specifically, word embedding-based models like FastText and PV-DBOW, which rely on word embeddings to capture semantic similarities, serve as fundamental approaches. While effective to some extent, our analysis indicates their limitations in fully capturing the complexities and contextual nuances present in diverse datasets, as evidenced by their performance. Sequence deep learning models, such as CNN and BiLSTM, which leverage the sequential nature of text, demonstrate improved performance, particularly in capturing context and long-range dependencies within text sequences. These findings underscore the significance of sequence modeling in text classification tasks.

Graph-based models, including our RB-GAT, represent the forefront of leveraging relational information embedded in text. These models exhibit superior performance, with RB-GAT achieving unprecedented accuracy rates: 71.48% on Ohsumed, 98.45% on R8, 80.32% on MR, 90.84% on 20NG and 95.67% on R52. This performance not only demonstrates RB-GAT’s effectiveness in diverse linguistic contexts but also highlights its innovative integration of GAT’s attention mechanism with the powerful language understanding capabilities of RoBERTa-BiGRU. RB-GAT’s leading edge can be attributed to its dual capacity to effectively model relational data through GAT and to deeply understand textual nuances via RoBERTa-BiGRU. This synergy enables RB-GAT to outperform both traditional models and contemporary graph-based approaches, positioning it as a significant advancement in text classification research.

Furthermore, and as detailed in Table 3, RB-GAT outperforms all considered models across the five datasets, with Macro F1 scores of 67.90% on Ohsumed, 94.84% on R8, 76.17% on MR, 85.43% on 20NG, and 77.25% on R52. This superior performance underscores the algorithm’s ability to effectively leverage the syntactic and semantic relationships within texts, attributed to the sophisticated integration of GAT’s attention mechanism with the contextual understanding capabilities of RoBERTa-BiGRU. Specifically, the Ohsumed dataset, with its medical terminologies and complex relationships, was notably well handled by RB-GAT, suggesting its potential in domains that require deep contextual understanding. On the R8 and MR datasets, RB-GAT’s performance further validates its generalizability and efficacy in capturing diverse textual phenomena.

#### 4.4.2. Comparison of the Accuracy of Models with Different Head Sizes

In our work, we utilized a multi-head graph attention network, wherein the number of heads functions as a hyperparameter. Each head corresponds to an independent attention model that influences the number of distinct attention mechanisms employed to compute node embeddings.

**Figure 2:**
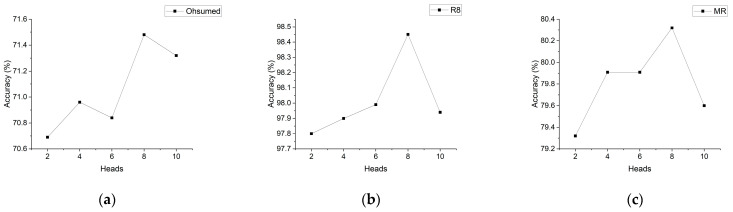
Comparison of classification accuracy of models with different head sizes for dataset: (**a**) Ohsumed, (**b**) R8, and (**c**) MR.

Figure 2 illustrates the classification accuracy of the RB-GAT model on the Ohsumed, R8, and MR datasets with varying head numbers. It is evident from the figure that the model’s accuracy varies depending on the chosen head numbers. Notably, on the Ohsumed, R8, and MR datasets, the RB-GAT model achieves its highest accuracy when the number of heads is set to 8, resulting in accuracies of 71.48%,98.45%, and 89.32%, respectively. This indicates that the use of eight heads provides an optimal balance between model complexity and performance, capturing intricate relationships within the data without introducing redundancy or noise. The overall trend observed across the three datasets is an initial increase followed by a subsequent decrease in accuracy. Specifically, for the Ohsumed dataset, an initial increase in the number of heads from 2 to 8 results in a gradual improvement in accuracy, peaking at 71.48% with 8 heads. This indicates an optimal density of attention mechanisms for capturing the intricate relationships within the biomedical literature categorized in Ohsumed. Conversely, a subsequent increase to 10 heads leads to a slight decrease in performance, suggesting a threshold beyond which additional heads may introduce noise or redundant information processing. This trend highlights a trade-off between model complexity and performance, where adding more heads beyond the optimal number can lead to overfitting or increased noise, negatively impacting model performance. Similar trends are observed for the R8 and MR datasets, where accuracy incrementally rises with the number of heads, reaching the highest accuracy of 98.45% and 80.32% with 8 heads, respectively.

In general, increasing the number of heads enhances the model’s expressive capability, enabling it to capture more intricate relationships among graph nodes. Additionally, a higher number of heads allows the model to focus on multiple aspects of the input graph concurrently, surpassing the limitations of a single attention mechanism. However, it is important to consider that augmenting the number of heads also increases the model’s complexity, leading to potential challenges during training and a higher risk of overfitting.

#### 4.4.3. Ablation Study

To investigate the contribution of each module in our model, we conducted an ablation study, the results of which are reported in Table 4. Specifically, “*w*/*o* RoBERTa” refers to a variant of RB-GAT that removes the RoBERTa module. “*w*/*o* BiGRU” is a variant that removes the BiGRU module. “*w*/*o* GAT” is a variant that replaces the GAT layer with a standard graph convolutional network (GCN) layer.

From Table 4, we can observe that RB-GAT achieves the best performance, with each variant also obtaining competitive results compared with other baseline models. Removing RoBERTa embeddings resulted in a noticeable drop in performance across all datasets, highlighting the critical importance of contextualized embeddings in capturing semantic nuances. The removal of the BiGRU layer also degraded performance, indicating that modeling sequential dependencies is crucial for understanding the order and relationships of words in sentences. Replacing GAT with a standard GCN significantly lowered accuracy, demonstrating the effectiveness of the attention mechanism in capturing important node relationships. The ablation study confirms that each component of RB-GAT significantly contributes to its overall performance. RoBERTa embeddings, BiGRU, and multi-head GAT each play a crucial role in enhancing the model’s ability to capture complex relationships and dependencies within the text.

## 5. Conclusions

In this paper, we introduce RB-GAT, a novel text classification model based on graph neural networks (GNNs) that combines the strengths of RoBERTa-BiGRU and a graph attention network. Our model is built upon the foundation of a heterogeneous graph, which captures intricate relationships between texts and words, utilizing advanced embedding techniques to represent these entities effectively. The integration of RoBERTa-BiGRU enables our model to preserve long-term dependencies and bidirectional information within texts, crucial for understanding the semantic richness of language. Additionally, the application of a multi-head, two-layer graph attention network allows RB-GAT to dynamically assess and weigh the importance of adjacent nodes in the graph, facilitating a deeper understanding of relationships and interactions within textual data.

The empirical evaluation of RB-GAT, performed on three well-established text classification datasets, highlights its superior performance compared with existing sequential deep learning and other GNN-based methods. These findings underscore the robustness, stability, and effectiveness of our model in tackling the challenges of text classification tasks. By providing a holistic solution that addresses both the representational and relational aspects of text data, RB-GAT establishes a new standard for future research in this field. We anticipate that the principles and methodologies presented in this paper will stimulate further advancements in text mining and related disciplines, pushing the boundaries of what can be achieved.

## Figures and Tables

**Table 1:** Summary statistics of datasets.

Datasets	#Docs	#Training	#Test	#Classes	#Nodes	#Edges	Average Length
Ohsumed	7400	3357	4043	23	21,557	8,066,963	135.82
R8	7674	5485	2189	8	15,362	3,504,462	65.72
MR	10,662	7108	3554	2	29,426	1,927,676	20.39
20NG	18,846	11,314	7532	20	61,603	26,990,597	221.26
R52	9100	6532	2568	52	17,992	4,406,322	69.82

**Table 2:** Test accuracy (%) comparison with baselines on benchmark datasets. (Note: “OOM” represents an out-of-memory error, and bold values indicate the best results).

Model	Ohsumed	R8	MR	20NG	R52
FastText	57.7	96.13	75.14	51.43	88.31
PV-DBOW	46.65	85.87	61.09	74.36	84.29
CNN	58.44	95.71	77.75	53.21	87.59
BiLSTM	49.27	96.31	77.68	73.18	90.54
TextGCN	68.36	97.07	76.74	86.34	93.56
SGC	68.53	97.23	75.91	91.14	93.07
Graph-CNN	63.86	96.99	77.22	81.42	92.75
TextING	70.42	98.04	79.82	OOM	93.65
TensorGCN	70.11	98.04	77.91	87.74	95.05
TextSSL	70.71	96.01	76.92	90.43	94.31
MHGAT	66.64	97.65	77.20	90.68	94.78
RB-GAT	**71.48**	**98.45**	**80.32**	**90.84**	**95.67**

**Table 3:** Test Micro F1 Score (%) comparison with baselines on benchmark datasets. (Note: “OOM” represents an out-of-memory error, and bold values indicate the best results).

Model	Ohsumed	R8	MR	20NG	R52
FastText	54.88	90.64	76.22	52.75	84.43
PV-DBOW	43.07	81.31	57.81	71.42	74.55
CNN	53.16	88.76	75.60	80.65	83.12
BiLSTM	48.66	88.55	75.26	73.18	83.06
TextGCN	61.45	92.88	75.58	81.34	72.56
SGC	65.34	93.50	71.90	84.54	74.33
Graph-CNN	59.49	92.90	74.04	78.13	73.11
TextING	66.51	93.85	75.41	OOM	74.65
TensorGCN	66.78	93.99	73.52	84.74	71.05
TextSSL	64.75	93.54	74.53	85.49	64.25
MHGAT	66.64	94.12	77.2	85.21	76.74
RB-GAT	**67.90**	**94.84**	**76.17**	**85.43**	**77.25**

**Table 4:** Ablation study of the RB-GAT over benchmark datasets in accuracy (%). (Note: Bold values indicate the best results).

Model	Ohsumed	R8	MR	20NG	R52
*w*/*o* RoBERTa	70.44	97.32	80.05	89.13	93.12
*w*/*o* BiGRU	71.02	97.14	79.46	89.66	93.67
*w*/*o* GAT	70.17	98.03	79.77	89.12	94.35
RB-GAT	**71.48**	**98.45**	**80.32**	**90.84**	**95.67**

## Data Availability

The data presented in this study are available on request from the corresponding author.
